# Genomic characteristics and comparative genomics analysis of the endophytic fungus *Sarocladium brachiariae*

**DOI:** 10.1186/s12864-019-6095-1

**Published:** 2019-10-28

**Authors:** Yang Yang, Xiaobao Liu, Jimiao Cai, Yipeng Chen, Boxun Li, Zhikai Guo, Guixiu Huang

**Affiliations:** 1 Environment and Plant Protection Institute, Chinese Academy of Tropical Agricultural Sciences, 4 Xueyuan Road, Haikou, 571101 China; 2 Institute of Tropical Bioscience and Biotechnology, Chinese Academy of Tropical Agricultural Sciences, 4 Xueyuan Road, Haikou, 571101 China

**Keywords:** *Sarocladium brachiariae*, Comparative genomics, CAZymes, Secondary metabolism, Gene cluster

## Abstract

**Background:**

*Sarocladium brachiariae* is a newly identified endophytic fungus isolated from *Brachiaria brizantha*. A previous study indicated that *S. brachiariae* had antifungal activity; however, limited genomic information restrains further study. Therefore, we sequenced the genome of *S. brachiariae* and compared it with the genome of *S. oryzae* to identify differences between a *Sarocladium* plant pathogen and an endophyte.

**Results:**

In this study, we reported a gapless genome sequence of a newly identified endophytic fungus *Sarocladium brachiariae* isolated from *Brachiaria brizantha*. The genome of *S. brachiariae* is 31.86 Mb, with a contig N50 of 3.27 Mb and 9903 protein coding genes. Phylogenomic analysis based on single copy orthologous genes provided insights into the evolutionary relationships of *S. brachiariae* and its closest species was identified as *S. oryzae*. Comparative genomics analysis revealed that *S. brachiaria* has 14.9% more plant cell wall degradation related CAZymes to *S. oryzae*, and 33.3% more fungal cell wall degradation related CAZymes, which could explain the antifungal activity of *S. brachiaria*. Based on Antibiotics & Secondary Metabolite Analysis Shell (antiSMASH) analysis, we identified a contact helvolic acid biosynthetic gene cluster (BGC) for the first time in *S. oryzae*. However, *S. brachiaria* had seven fewer terpene gene clusters, including helvolic acid BGC, compared with *S. oryzae* and this may be associated with adaptation to an endophytic lifestyle. Synteny analysis of polyketide synthases (PKS), non-ribosomal peptide synthetases (NRPS), and hybrid (PKS-NRPS) gene clusters between *S. brachiariae* and *S. oryzae* revealed that just 37.5% of tested clusters have good synteny, while 63.5% have no or poor synteny. This indicated that the *S. brachiariae* could potentially synthesize a variety of unknown-function secondary metabolites, which may play an important role in adaptation to its endophytic lifestyle and antifungal activity.

**Conclusions:**

The data provided a better understanding of the *Sarocladium brachiariae* genome. Further comparative genomic analysis provided insight into the genomic basis of its endophytic lifestyle and antifungal activity.

## Introduction

The *Sarocladium* genus was firstly established in 1976 based on two fungal pathogens causing sheath rot of rice [1]. Based on rDNA and internal transcribed spacer (ITS) sequences, some *Acremonium* species were recently relocated to the genus *Sarocladium*. Most *Sarocladium* species are plant and human pathogens, such as *S. oryzae*, *S. kiliense*, and *S. strictum*, and include a maize endophyte, *S. zeae* [2, 3]. In previous study, we isolated an endophytic fungus strain HND5 from healthy leaves of *Brachiaria brizantha* collected form Danzhou China. This fungus has raised, cottony and moist to slimy colonies on PDA and produces branching conidiophore, cylindrical conidia arranged in slimy heads. Key morphological feature of the fungi is the production of hyphal coil. Together with ITS and LSU rDNA sequence phylogenetic analysis, we proposed it as *Sarocladium brachiariae* (MycoBank no. 814539), a new species of *Sarocladium* [4]. This new species prominently shows broad-spectrum inhibition to growth of many tested plant pathogenic fungi on solid media plate, including *Colletotrichum gloeosporioides* of mango trees, *Fusarium oxysporium* f.sp. *cubense*, *Gloeosporium musarum*, *Colletotrichum gloeosporioides* of rubber trees, *Corynespora cassicola* of papaya, *Fusarium oxysporium* of bamboo, *Magnaporthe grisea*, *Bipolaris oryzae* Shoem, *Colletotrichum falcatum* Went and Drechslera sp. We also observed *S. brachiariae* could colonize inside root of banana using GFP-tagging. And colonization of *S. brachiariae* could reduce the incidence of banana fusarium wilt in the field [5]. The genomic resources of *Sarocladium* in public database are limited and just whole genome of phytopathogenic species *S. oryzae* has been sequenced and published [6]. In order to unravel the genomic basis of endophytic lifestyle and antifungal activity of *S. brachiariae*, we sequenced the whole genome of *S. brachiariae* and made a comparative analysis with genome of *S. oryzae*.

Carbohydrate-active enzymes (CAZymes) are responsible for the breakdown, biosynthesis or modification of glycoconjugates, oligo- and polysaccharides [7]. Fungi can produce all kinds of CAZymes and Hittalmani et al. identified 1042 glycoside hydrolases (GHs), 1115 glycosyltransferases (GTs), 416 carbohydrate esterases (CEs), 270 auxillary activities (AAs) and 11 polysaccharide lyases (PLs) from genome of *S. oryzae* Saro-13 strain [6]. Secreted CAZymes involved in plant cell wall or fungal cell wall degradation received special attention because of their importance in phytopathogenic and endophytic fungi penetration of their hosts or biocontrol fungi inhibition of target pathogenic fungi. To overcome plant cell wall to colonize, plant pathogenic and endophytic fungi produce various enzymes to deconstruct cell well polysaccharides and these enzymes are called “cell wall-degrading enzymes (CWEDs)” [8, 9]. The CAZy database (Carbohydrate Active Enzymes database, http://www.cazy.org/) has classified CWEDs and divided them into different families [7]. Enzymes involved in cellulose and hemicellulose hydrolysis are distribute mainly in the glycoside hydrolase (GH) families [10]. As pectin degradation requires polygalacturonidases and pectin/pectate lyases, CWEDs involved in pectin hydrolysis are classified into GH 28 and polysaccharide lyases (PL) families [11]. In contrast to the plant cell wall, the fungal cell wall is mainly composed by chitin and β-(1,3)-glucan. Thus, secreted chitinases and β-(1,3)-glucanases are responsible for fungal cell wall degradation [12]. According to the CAZy classification, enzymes involved in chitin degradation mainly belong to GH18 and GH75 families, and the enzymes responsible for β-(1,3)-glucan could be found in the GH55, GH16 and GH81 families [10, 13]. Compared with other species, fungi with antifungal activity usually contain expanded CAZyme families involved in fungal cell wall degradation, for example in *Trichoderma atroviride* and *Trichoderma virens* [14].

Secondary metabolites (SMs) are defined as bioactive, small molecules that are not essential to the growth of an organism [15]. Studies of SMs in *Sarocladium* genus have mainly focused on phytotoxins, as most species are plant pathogens. Two phytotoxins, helvolic acid and cerulenin, have been detected in liquid culture of *S. oryzae*, the pathogen causing rice sheath-rot, and were also found in infected rice sheath tissues [16, 17]. Helvolic acid is a tetracyclic triterpenoid and can affect chlorophyll biosynthesis. Cerulenin is an epoxydodecadienamide that can inhibit polyketide and fatty acid synthesis by inhibiting malonyl-ACP:acyl-ACP condensation [18]. The biosynthetic pathways of helvolic acid have been elucidated in *Aspergillus flavus* and *Metarhizium anisophilae* [19, 20]. When the *S. oryzae* genome was sequenced, Hittalmani et al. identified nine candidate genes involved in the helvolic acid biosynthesis pathway, based on protein homology analysis in *S. oryzae* [6].

Besides biosynthetic gene cluster (BGC) of helvolic acid, *S. oryzae* also contains other kinds of BGCs with unknown function, such as such as polyketide synthases (PKS), non-ribosomal peptide synthetases (NRPS) and hybrids (PKS-NRPS) [6]. NRPSs and PKSs are both large, multi-modular enzymes. NRPS modules contain three primary functional domains: Adenylation (A), thiolation (T), and condensation (C) [21]. PKS modules usually contain ketosynthase (KS), malonyl-CoA:acyl carrier protein transacylase (MAT), acyl carrier protein (ACP), ketoreductase (KR), and dehydratase (DH) [22]. Their high level of amino acid and nucleotide conservation mean that the A domains and KS domains are frequently used to reconstruct the evolutionary histories of NRPS and PKS, respectively [23, 24]. Backbone biosynthetic genes are often clustered with different kinds of enzyme-coding genes, such as cytochrome P450, methyltransferase, and hydroxylase. Usually, BGCs are highly distinct, and even between similar fungi whose genomes exhibit high sequence and synteny conservation, the identity and total number of BGCs can vary widely [25]. Currently, genomic studies are the best way to obtain a global view of fungal BGCs and comparative genomics allows the analysis of a fungus’ potential to produce SMs [26].

In the present study, we report a gapless genome sequence of *S. brachiariae*, an endophytic fungus isolated from *Brachiaria brizantha*, which has the prospect of being applied as a biocontrol-agent. To understand the important pathways and genes utilized by *S. brachiariae* to carry out its antifungal and endophytic activities, we compare its genome with that of *S. oryzae*. After a general genome comparison, this study focused on comparing genes involved in CAZymes and SM biosynthesis. The results showed that *S. brachiariae* has more CAZymes involved in plant cell wall degradation and more CAZymes involved in fungi cell wall degradation, compared with those in *S. oryzae*. Based on Antibiotics & Secondary Metabolite Analysis Shell (antiSMASH) analysis, we identified a contact helvolic acid biosynthetic gene cluster (BGC) for the first time in *S. oryzae*. In addition, antiSMASH analysis result also indicated that *S. brachiaria* had seven fewer terpene gene clusters, including the helvolic acid BGC, compared with those in *S. oryzae*. Synteny analysis of PKS, NRPS, and PKS/NRPS gene clusters between *S. brachiariae* and *S. oryzae* revealed that only 37.5% of the tested clusters have good synteny, while 63.5% have no or poor synteny. This indicated that the *S. brachiariae* could potentially synthesize a variety of unknown-function secondary metabolites, which may play an important role in adaptation to its endophytic lifestyle and antifungal activity. The information contained in this study will be helpful to understand the biological nature of the endophytic fungus *S. brachiariae* and will widen our understanding of the *Sarocladium* genus.

## Results

### General genome features and annotation

The genome of *S. brachiariae* was sequenced to 191.0× coverage using a newly developed Single Molecule Real-Time (SMRT) sequencing technique, which can assist in obtaining gapless eukaryotic genome sequences [27]. CANU was used for de novo assembly of the sequencing data (6,115,460,666 bp clean reads), which generated 19 contigs with a N50 length of 3.27 Mb (Table 1). The genome assembly comprises approximately 31.86 Mb, which is slightly smaller than that of *S. oryzae* (32.40 Mb, GCA_001972265.1), and both of them are smaller than the average genome size of the Ascomycota (36.91 Mb) [6, 28]. The completeness of the genome assembly was assessed using BUSCO (benchmarking universal single-copy Orthologs; https://busco.ezlab.org/), which showed that 1287 out of the 1375 (97.8%) gene groups required for the correct assembly of Ascomycota are present in the *S. brachiariae* scaffolds assembly (Additional file 2: Table S1.1). Total RNA of *S. brachiariae* was extracted from mycelia collected from both PDA plates and PDB culture inoculated for 7 days. We got 93,212,574 clean reads and 13,889,709,506 clean bases using Illumina novaseq 6000 system (Additional file 2: Table S1.2). TopHat2 analysis result indicated 89.4% genome pairs of *S. brachiariae* could be mapped with RNAseq result (Additional file 2: Table S1.3). Besides RNAseq, 22 genes from *S. brachiariae* were chosen for PCR validation of gene prediction result and all these genes could be amplified from genome DNA of *S. brachiariae* (Additional file 1: Figure S1; Additional file 8). These findings suggested that this assembly and in silico gene prediction is intact and robust.

**Table 1:** Genome characteristics and assembly features of *S. brachiariae* and *S. oryzae*

Genome	*S. brachiariae* HND5	*S. oryzae* IMI 501407
Total genome size (bp)	31, 862,451	32,403,370
Number of contigs	19	215
Total assembly gap length	0	64,323
Depth of genome coverage (x)	191	336
N50 value (bp)	3,269,626	322,791
G + C content (%)	52.04	53.1

Compared with *S. oryzae* (53.1%), *S. brachiariae* has a lower GC content genome (52.04%) (Table. 1).The *S. brachiariae* genome contains 55 rRNAs, 24 snRNAs, 1 miRNA, and 78 tRNAs (Additional file 2: Table S2). The de novo and reference based repeat analysis showed that 1.39% of genome is repetitive (Additional file 2: Table S3). A total 9903 protein-coding genes were predicted in the *S. brachiariae* genome, and 95.67% of predicted genes in *S. brachiariae* could be annotated. Among these predicted genes, 3801 (31.1%), 5443 (54.96%), 2999 (30.28%), 9464 (95.57%), and 6919 (69.87%) could be annotated based on Clusters of Orthologous Groups (COG), gene ontology (GO), Encyclopedia of Genes and Genomes (KEGG), the non-redundant protein database (NR), and Swiss-Prot (Additional file 2: Table S4).

### Orthology analysis of *S. brachiariae* and another five Sordariomycetes fungi

**Fig. 1:**
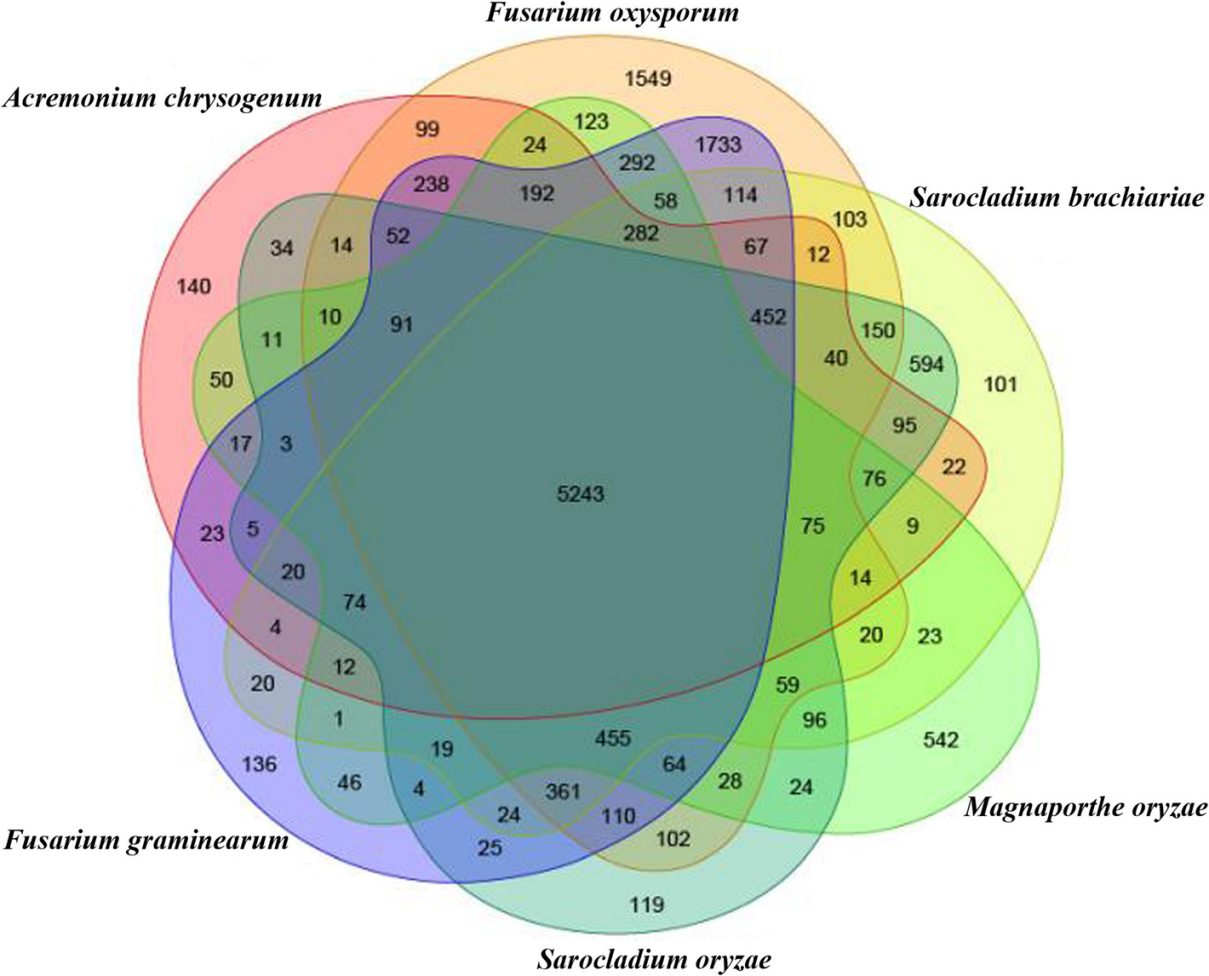
Venn diagram showing shared and distinct orthologous gene families in Sordariomycetes fungi. The proteomes of *Fusarium oxysporum*, *F. graminearum*, *S. oryzae*, *S. brachiariae*, *Acremonium chrysogenum*, and *Magnaporthe oryzae* were clustered using orthoMCL. The numbers of genes in each species are shown in parenthesis

Orthology is a very efficient way to identify differences and similarities between model organisms and uncharacterized genomes [29]. An OrthoMCL analysis was performed on *S. brachiariae* and five other Sordariomycetes fungi (*Fusarium oxysporum*, *F. graminearum*, *S. oryzae*, *Acremonium chrysogenum*, and *Magnaporthe oryzae*) with known genomes. A Venn diagram of the OrthoMCL analysis showed that *S. brachiariae* shares 5243 genes with the other five Sordariomycetes fungi (Fig. 1). The two *Sarocladium* species shared similarly low number of unique genes, *S. brachiariae* harbored the lowest number (101) of unique genes and *S. oryzae* harbored 119 unique genes in this group: *F. oxysporum*, *F. graminearum*, *A. chrysogenum*, and *M. oryzae* possessed more unique genes (1553, 134, 140, and 542 respectively).

### Phylogenetic relationship

**Fig. 2:**
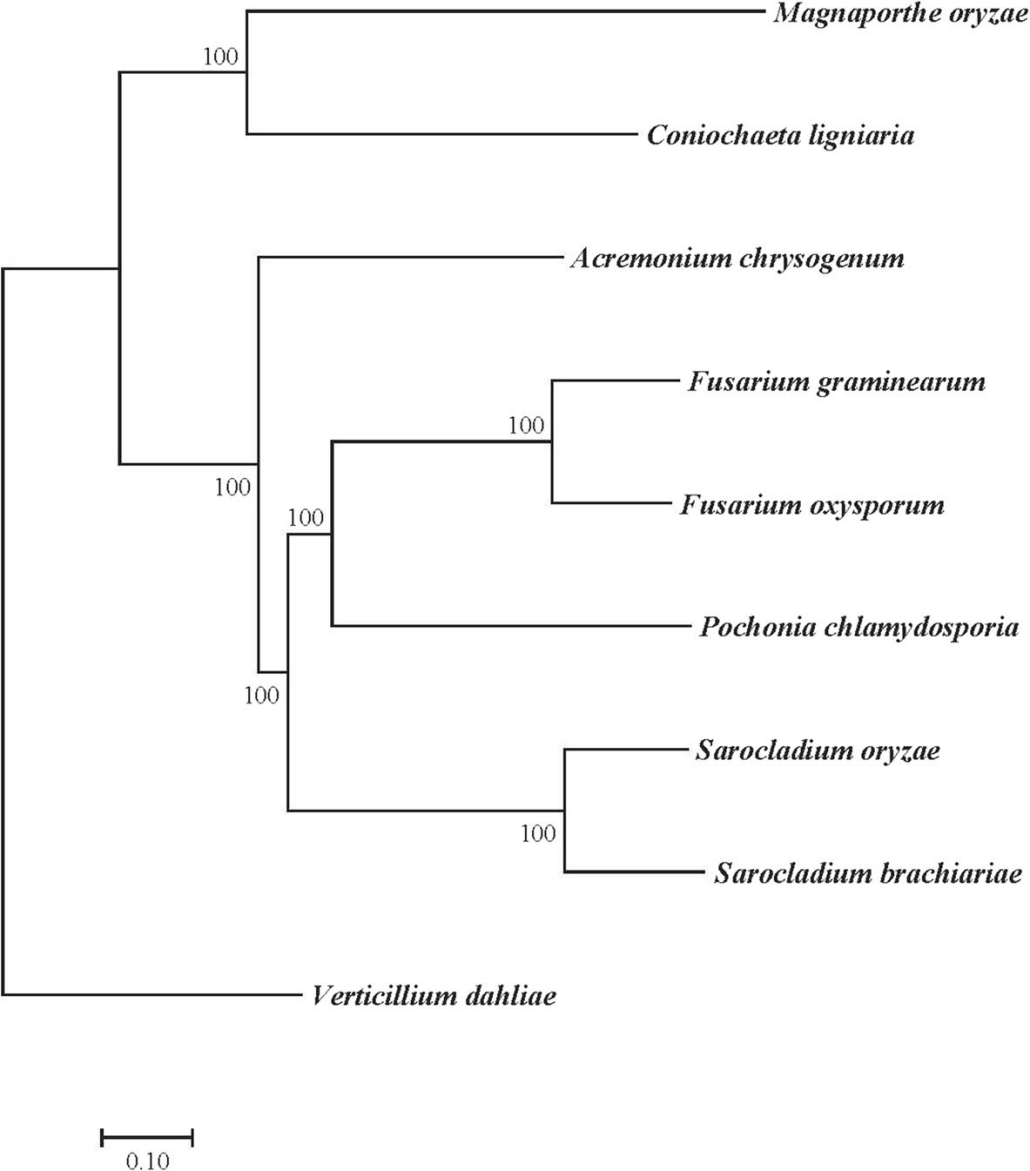
Maximum Likelihood phylogenomic tree based on single copy orthologous genes

Based on *Sarocladium* phylogenetic relationship with other Sordariomycetes fungi [2, 3], seven Sordariomycetes genomes, including two endophytic fungi (*Pochonia chlamydosporia* and *Coniochaeta ligniaria*), five plant pathogens (*Magnaporthe grisea*, *Verticillium dahliae*, *F. oxysporum*, *F. graminearum*, and *S. oryzae*), and 1 marine fungus (*A. chrysogenum*), were used for phylogenomic analysis with *S. brachiariae*. Single-copy orthologous proteins were used to build the phylogenetic tree. The Maximum Likelihood phylogeny tree was generated by the RaxML [30] method based on the GTRGAMMA model. The result revealed that *S. brachiariae* was evolutionarily close to *S. oryzae*, a plant pathogen that causes sheath rot disease on rice (Fig. 2) [6]. In addition, *S. brachiariae* was also close to the other two plant pathogens, *Fusarium oxysporum* and *F. graminearum*, and one endophytic fungus *Pochonia chlamydosporia* (Fig. 2).

### Functional annotation

**Fig. 3:**
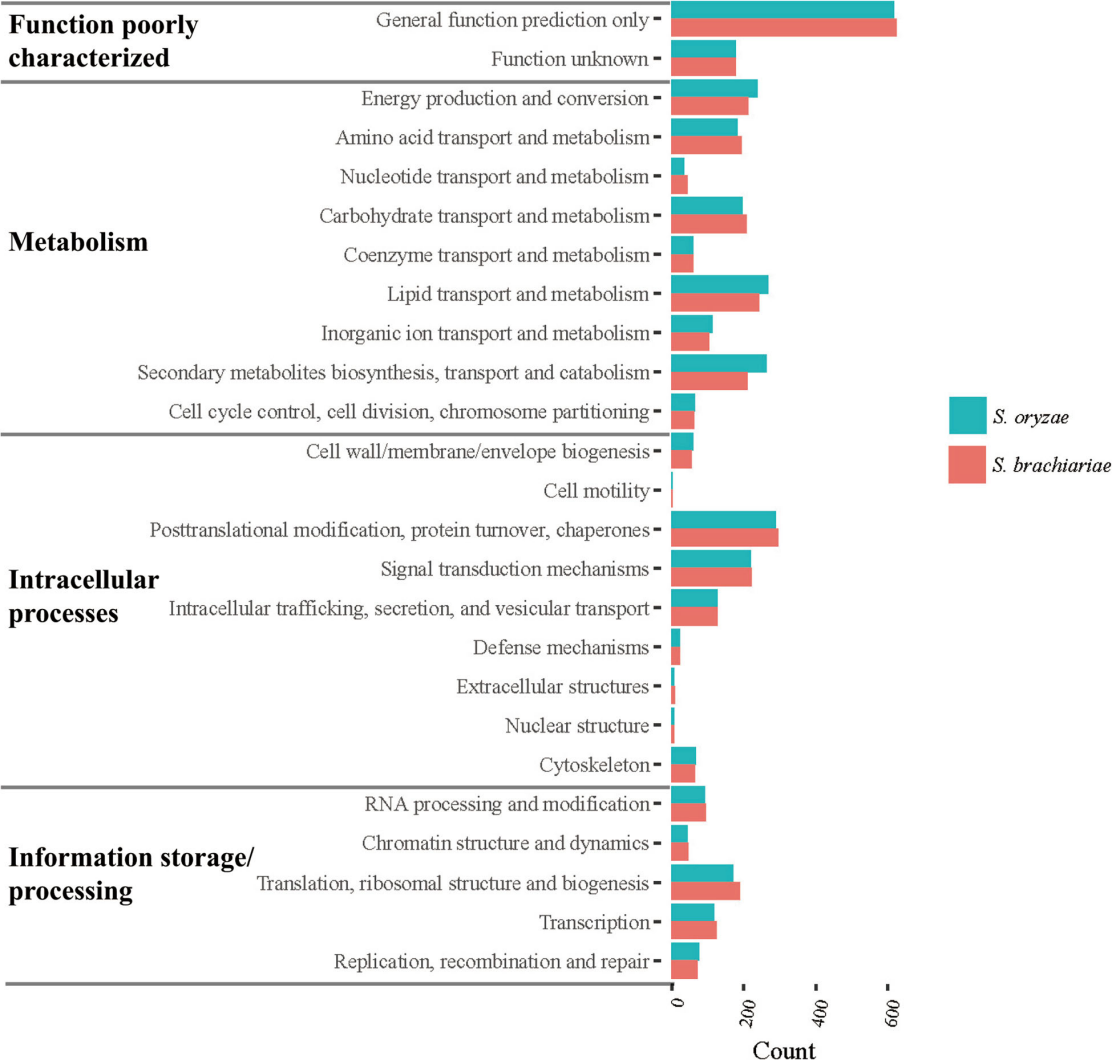
Histogram of KOG distribution of predicted proteins from *S. brachiariae* and *S. oryzae*

Using euKaryotic Orthologous Groups (KOG) [31] functional classification, we assigned functions to proteins based on sequence similarity (Fig. 3; The raw data are available in Additional file 2: Table S5). Of the total predicted proteins, 3466 *S. brachiariae* proteins and 3516 *S. oryzae* proteins had KOG hits. These were classified into four main groups: Function poorly characterized, Metabolism, Intracellular processes, and Information storage/processing. KOG analyses revealed that the global pattern of protein allocation of *S. brachiariae* was very similar to that *S. oryzae*, indicating that these two strains are closely related and might have similar ecological niches. However, in one category, “Secondary metabolites biosynthesis, transport and catabolism”, *S. brachiariae* had fewer hits (209) than *S. oryzae* (262), which indicated the secondary metabolites of these two species might be very different.

### Whole-genome synteny comparisons between *S. brachiariae* and *S. oryzae*

**Fig. 4:**
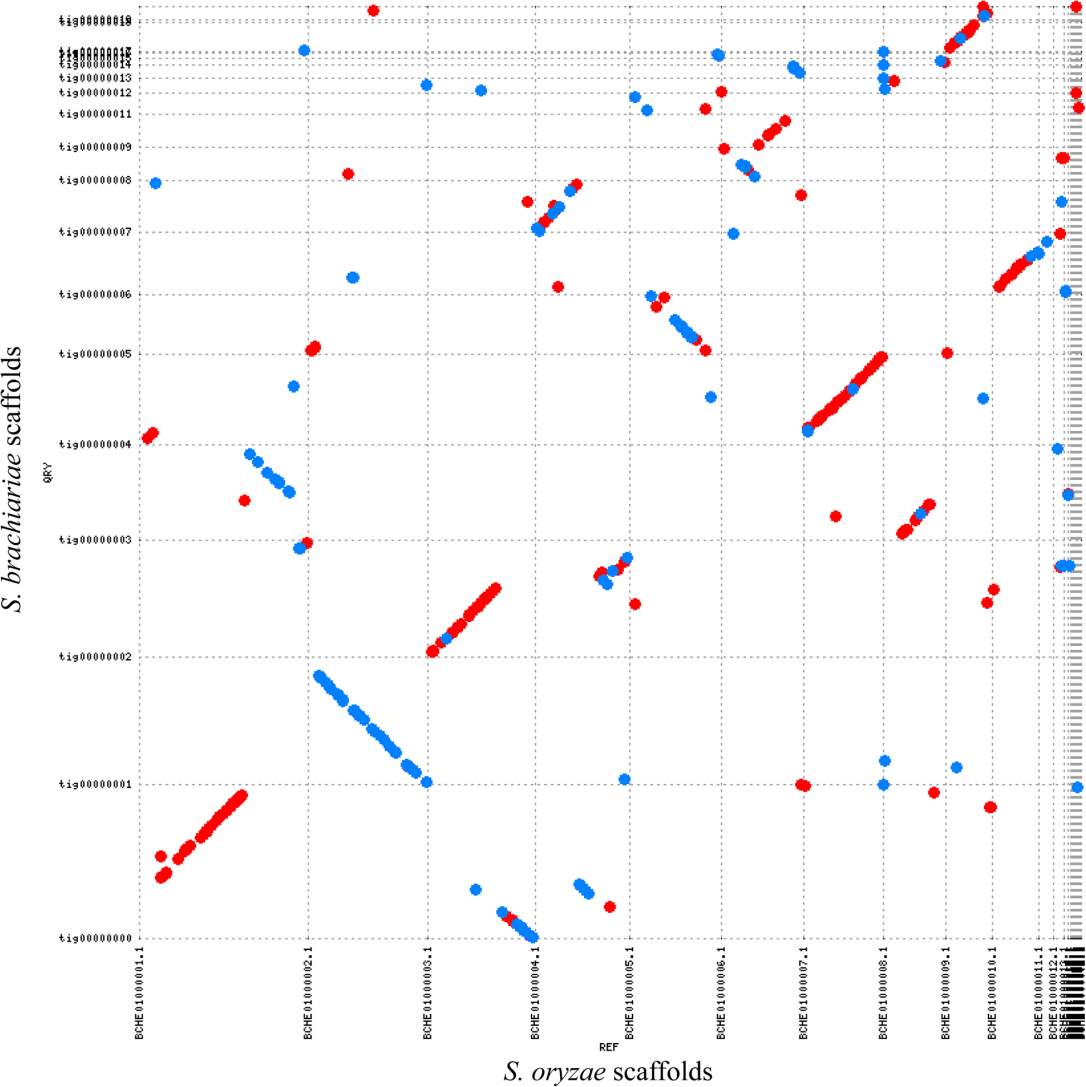
Genome synteny analysis between *S. brachiariae* and *S. oryzae*

The phylogenomic analysis revealed that *S. brachiariae* is evolutionarily close to *S. oryzae*; therefore, we performed synteny comparison between these two species. The MUMmer software was used to do the analysis and synteny dot-plot was generated using mummerplot [32]. The generated synteny dot-plot showed the macrosynteny between the two genomes and high levels of sequence homology to each other with more than 95% sequence identity (Fig. 4). Especially, contigs 0, 1, 2, 4, 5, and 6 of *S. brachiariae* corresponded well with contigs 1, 2, 3, 7, 5, and 10 of *S. oryzae* (Fig. 4). 25.81% of *S. brachiariae* genome and 24.49% of *S. oryzae* gnome shared high synteny, indicating these two *Sarocladium* species share conserved and core genes.

### Carbohydrate-active enzymes

**Fig. 5:**
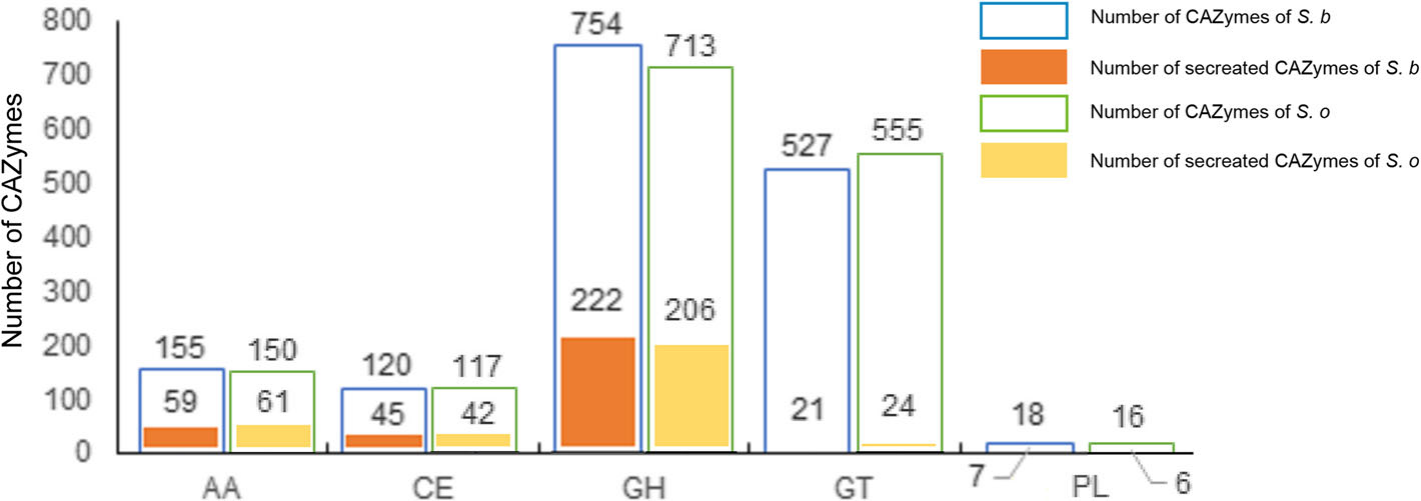
Distribution of CAZymes and secreted CAZymes in *S. brachiariae* and *S. oryzae*

Carbohydrate-Active Enzymes (CAZymes) are crucial for fungal biological activity. For plant pathogens and endophytes, CAZymes are responsible for degradation of the host plant cell and to establish colonization. In addition, biocontrol fungi can use CAZymes to destroy the cell walls of pathogens and nematodes [10, 13]. Using the Carbohydrate Active Enzymes database, we annotated 1574 CAZyme proteins distributed across 155 CAZyme families in the *S. brachiariae* genome and 1551 CAZyme proteins distributed across 154 CAZyme families in *S. oryzae* genome. Among all CAZymes, 11.9% (187) in *S. brachiariae* and 10.8% (167) in *S. oryzae* are secreted enzymes (Fig. 5; Additional file 2: Table S6). CAZymes were further classified by catalytic activity: Auxiliary activities (AAs), carbohydrate esterases (CEs), glycoside hydrolases (GHs), glycosyl transferases (GTs), and polysaccharide lyases (PLs). As shown in Fig. 5, CAZymes and secreted CAZymes from *S. brachiariae* and *S. oryzae* had a similar distribution. These two compared strains are plant associated fungi and *S. brachiariae* has antifungal activity; therefore, we analyzed the differences between CAZymes involved in plant and fungal cell wall degradation. According to the classification of Zhao [10] and Kubicek [8], CAZymes involved in plant cell wall degradation, such as cellulose, hemicellulose and pectin degradation, are listed in Table 2. CAZymes involved in chitin and β-1,3-glucan degradation, which are major components of the fungal cell wall [13], are also listed in Table 3. The result of the comparison indicated that *S. brachiariae* possessed 14.9% more plant cell wall degradation CAZymes than *S. oryzae*. For the CAZymes involved in fungal cell wall degradation, *S. brachiariae* had 33.3% more of secreted CAZymes than *S. oryzae*. This result indicated that *S. brachiariae* might have better fungal cell wall degradation ability than *S. oryzae*.

**Table 2:** CAZymes involved in plant cell wall degradation

Substrates	Family	*S. brachiariae*		*S. oryzae*	
		Copy number	Secreated copy number	Copy number	Secreated copy number
Cellulose	GH1	4	1	5	1
	GH3	29	4	27	5
	GH5	20	8	23	9
	GH12	14	3	8	3
	GH45	1	1	2	2
	GH6	4	3	4	2
	GH7	7	7	7	7
	GH74	1	1	1	1
hemicellulose	GH10	5	3	6	2
	GH11	10	9	10	9
	GH30	11	3	9	1
	GH115	2	0	1	0
	GH27	6	3	6	2
	GH29	0	2	4	2
	GH35	3	1	3	1
	GH36	16	0	13	0
	GH39	0	2	7	3
	GH51	2	0	2	0
	GH53	2	2	1	1
	GH54	6	4	3	3
	GH62	3	3	1	1
	GH67	2	1	2	1
	GH93	6	1	6	1
	GH43	44	12	45	9
Pectin	GH105	2	1	4	1
	GH28	16	3	16	2
	GH78	10	0	6	0
	GH88	2	0	2	0
	PL1	6	4	6	3
	PL3	2	2	2	2
	PL4	7	1	5	0
Total number		243	85	237	74

Abbreviations: GH, glycoside hydrolase; PL, polysaccharide lyase

**Table 3:** CAZymes involved in fungal cell wall degradation

Substrates	Family	*S. brachiariae*		*S. oryzae*	
		Copy number	Secreated copy number	Copy number	Secreated copy number
Chitin	GH18	132	17	127	13
	GH20	9	0	8	0
	GH75	4	1	4	1
	GH76	20	0	20	0
β-1,3-glucan	GH17	21	1	21	2
	GH55	11	1	11	2
	GH71	4	0	5	0
	GH72	8	0	8	0
	GH81	3	0	2	1
	GH16	65	16	47	8
Total number		277	36	253	27

Abbreviations: GH, glycoside hydrolase

### Secondary metabolite gene clusters

By definition, SMs are small bioactive molecules that were not strictly necessary for growth and development of an organism [25]. However, SMs are important for fungi’s lifestyle, such as the antibiotics produced by biocontrol fungi and the phytotoxins synthesized by plant pathogens. The genes required for SM synthesis are usually arranged in a multigene biosynthetic gene cluster in fungi [33]. To analyze the SM synthesis potential of *S. brachiariae*, we used antiSMASH 3.0 (fungi view) to identify gene clusters in the genome of *S. brachiariae*. We also analyzed the genome of *S. oryzae* (GenBank: BCHE00000000.1) for comparison. The list of *S. brachiariae* and *S. oryzae* putative SM clusters and their genomic coordinates were shown in Additional file 2: Table S7.

As shown in Table 4, *S. brachiariae* has 34 SM gene clusters, including 7 non-ribosomal peptide synthase (NRPS) clusters, 12 polyketide synthase (PKS) clusters, 5 PKS/NRPS clusters, 6 terpene synthase clusters, and 4 other clusters. Compared with *S. brachiariae*, *S. oryzae* had 4 fewer PKS cluster, 6 more PKS/NRPS clusters, and 7 more terpene synthase clusters.

**Table 4:** The secondary metabolites gene clusters in *S. brachiariae* and *S. oryzae*

Genome	*S. brachiariae*	*S. oryzae*
NRPS	7	7
PKS	11	8
PKS-NRPS	5	10
Terpene	6	13
Other	5	8
Total	34	46

Abbreviations: NRPS, nonribosomal peptides synthase; PKS, polyketide synthase; PKS-NRPS: hybrid NRPS-PKS enzymes

### Helvolic acid biosynthetic gene cluster analysis in *S. brachiariae* and *S. oryzae*

**Fig. 6:**
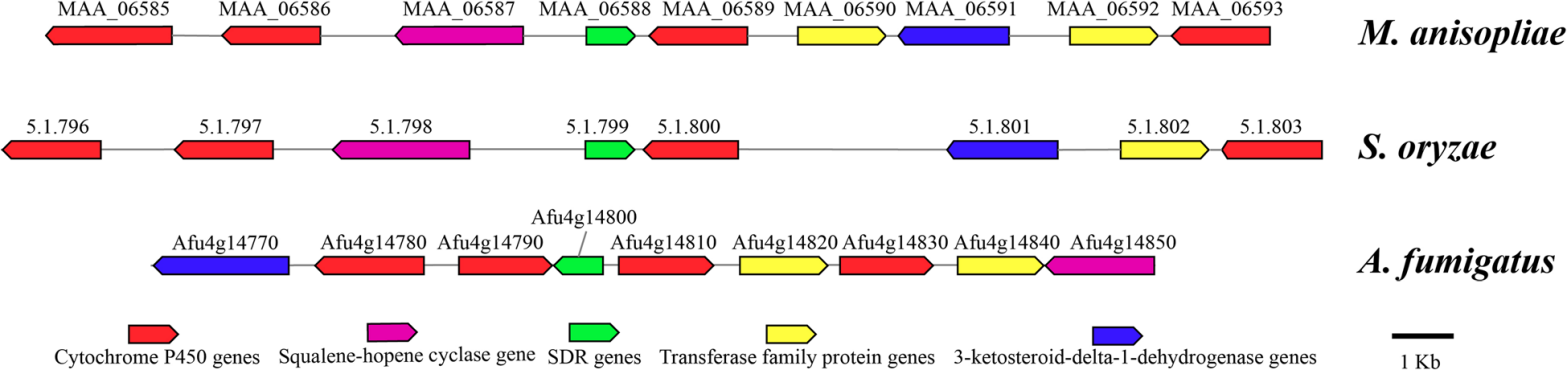
Organization of putative gene clusters involved in helvolic acid biosynthesis in *S. oryzae*

Helvolic acid and cerulenin were the two main phytotoxic metabolites synthesized and secreted by the rice pathogen *S. oryzae* [6, 34]. As an initial event in pathogenesis, helvolic acid and cerulenin can alter membrane permeability and cause electrolyte leakage [16]. The biosynthesis pathway for helvolic acid has been elucidated in *Aspergillus fumigatus* Af293 [19, 35]. There are nine genes in the helvolic acid synthesis cluster, including an oxidosqualene cyclase (OSC), a short-chain dehydrogenase/reductase (SDR), a 3-ketosteroid-Δ1-dehydrogenase (KSTD), two acyltransferases, and four cytochrome P450s (CYP5081 family). Based on BLASTN search results, Hittalmani reported that nine genes are involved in helvolic acid biosynthesis in *S. oryzae* Saro-13 strain [6]. However, these nine genes spread across the whole genome and did not form a biosynthetic gene cluster (BGC). In the present study, using antiSMASH, we located a gene cluster in *S. oryzae* JCM 12450 strain that shared 77% similarity with the helvolic acid BGC reported in *A. fumigatus* Af293 (MIBiG BGC: BGC0000686). We further annotated genes in this cluster, and found this cluster had a similar organization to the helvolic acid BGC reported in *M. anisophilae*. However, the newly identified helvolic acid BGC only contained eight genes and was lacking one of the acyltransferase genes (Fig. 6, Additional file 3: Table S1). Therefore, this is a new type BGC for helvolic acid synthesis and is the first intact helvolic acid BGC identified in *Sarocladium* genus.

The antiSMASH result of *S. brachiariae* did not identify any cluster involved in helvolic acid synthesis. Among the genes responsible for helvolic acid biosynthesis, SDR, acyltransferase, and P450 genes are numerous in fungal genomes, while OSC and KSTD are rare. Therefore, we performed TBLASTN searches of the *S. brachiariae* genome against OSC and KSTD protein sequences (Afu4g14770, Afu4g14850) to locate the probable helvolic acid synthesis cluster. One OSC (WHWLZ9448) and one KSTD (WHWLZ3787) encoding gene were found in *S. brachiariae* genome. Fourteen genes around these two genes (within a distance of ≥10Kb) were further annotated; however, no further genes related to helvolic acid synthesis were found (Additional file 3: Table S2). In addition, a genome wide analysis for cytochrome P450 genes showed that *S. brachiariae* has 388 CYP genes, but none of them belong to the CYP5081 family (Additional file 2: Table S8), which is critical for helvolic acid synthesis [20]. Taken together, these results strongly suggest that *S. brachiariae* lacks the genome resources to synthesize helvolic acid. *S. brachiariae* is an endophyte and can coexist with host plants without causing any damage [4]. Therefore, the lack of the phytotoxin helvolic acid BGC in *S. brachiariae* might be an adaptation method for its endophytic lifestyle.

### Phylogenomic analysis of NRPS, PKS, and PKS/NRPS genes of *S. brachiariae* and *S. oryzae*

**Fig. 7:**
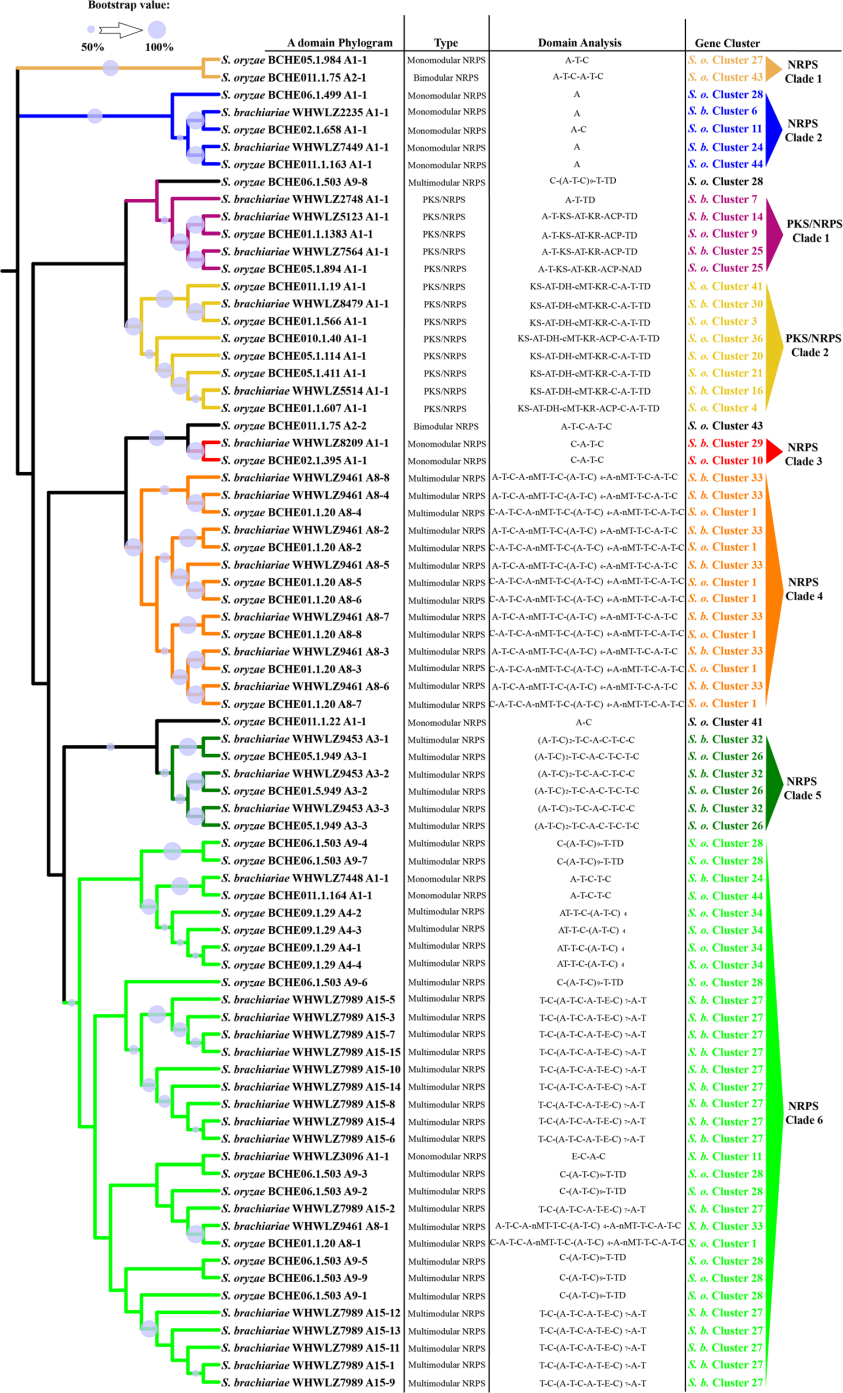
Phylogenetic analysis of NRPSs and PKS/NRPSs from *S. brachiariae* and *S. oryzae* based on the A domain sequence. The PKS and NRPS domains were determined based on antiSMASH analysis. The ML tree was generated using MEGA6 with the WAG model. Bootstrap support greater than 50% is shown on the branches. A domain sequences used in this analysis was listed in Additional file 6

**Fig. 8:**
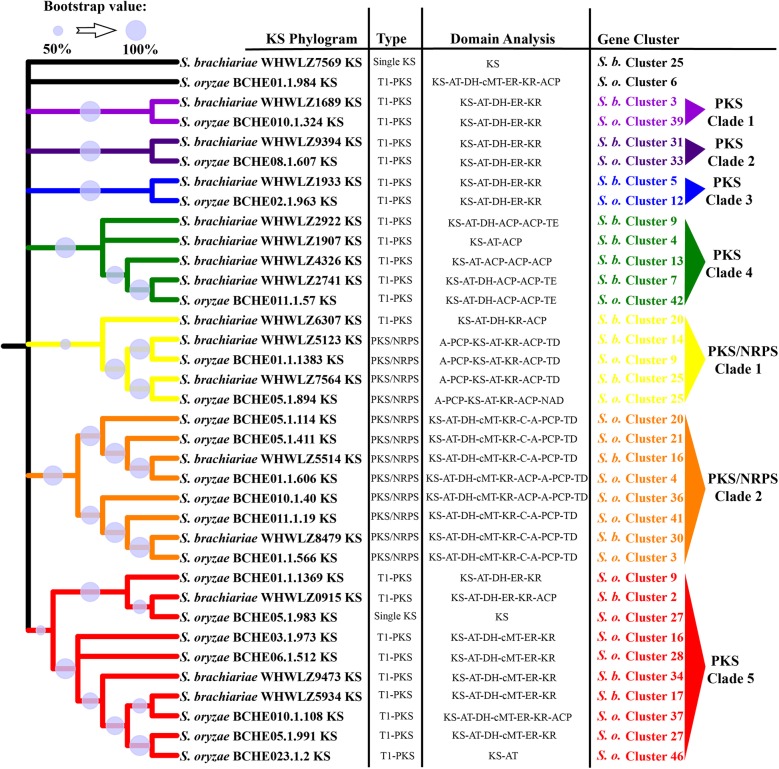
Phylogenetic analysis of PKSs and PKS/NRPSs from *S. brachiariae* and *S. oryzae* based on the KS domain sequence. The PKS and NRPS domains were determined based on antiSMASH analysis. The ML tree was generated using MEGA6 with the WAG model. Bootstrap support greater than 50% is shown on the branches. KS domain sequences used in this analysis was listed in Additional file 7

Many fungal SMs are synthesized by multimodular enzymes, NRPSs, PKSs, or hybrid PKS/NRPS. To determine differences between the secondary metabolomes of *S. brachiariae* and *S. oryzae*, we analyzed the phylogenomic relationships of NRPSs, PKSs, and PKS/NRPS identified in these two species. As A domain and KS domain are relatively conserved in NRPS and PKS [23, 36]; therefore, the phylogenetic relationships among NRPSs, PKSs, and PKS/NRPS were analyzed based on the A domain or KS domain sequences. Phylogenetic analysis of the A domains revealed that NRPSs from *S. brachiariae* and *S. oryzae* could be grouped into six clades, NRPS1–3 clades are mainly mono−/bimodular NRPSs and NRPS 4–6 clades are multimodular NRPSs (Fig. 7). The KS domain phylogenetic analysis indicated that the PKSs from the two compared strains could be grouped into five clades (Fig. 8). PKS/NRPS contain both A and KS domains; the PKS/NRPS phylogenetic relationships were analyzed using the A and KS domains, separately. As shown in Figs. 7 and 8, PKS/NRPSs from the two compared strains formed two clades and A domain and KS domain phylogenetic analysis showed the same result. Both clades had more than 50% bootstrap support. Further analysis of the domain structure revealed that synthases from the same clade shared a similar domain structure (Figs. 7 and 8). Most clades contain an equal number synthases from *S. brachiariae* and *S. oryzae*, such as NRPS clades 1, 3, 4 and 5; and PKS clade 1, 2, and 3. However, some clades contain unequal number of synthases, such as PKS clade 4, which contains one PKSs from *S. oryzae* and four PKSs from *S. brachiariae*. This indicated that high level gene duplication had happened during the evolution of SM-related genes in the *Sarocladium* genus.

### Synteny analysis of SM gene clusters of *S. brachiariae* and *S. oryzae*

**Fig. 9:**
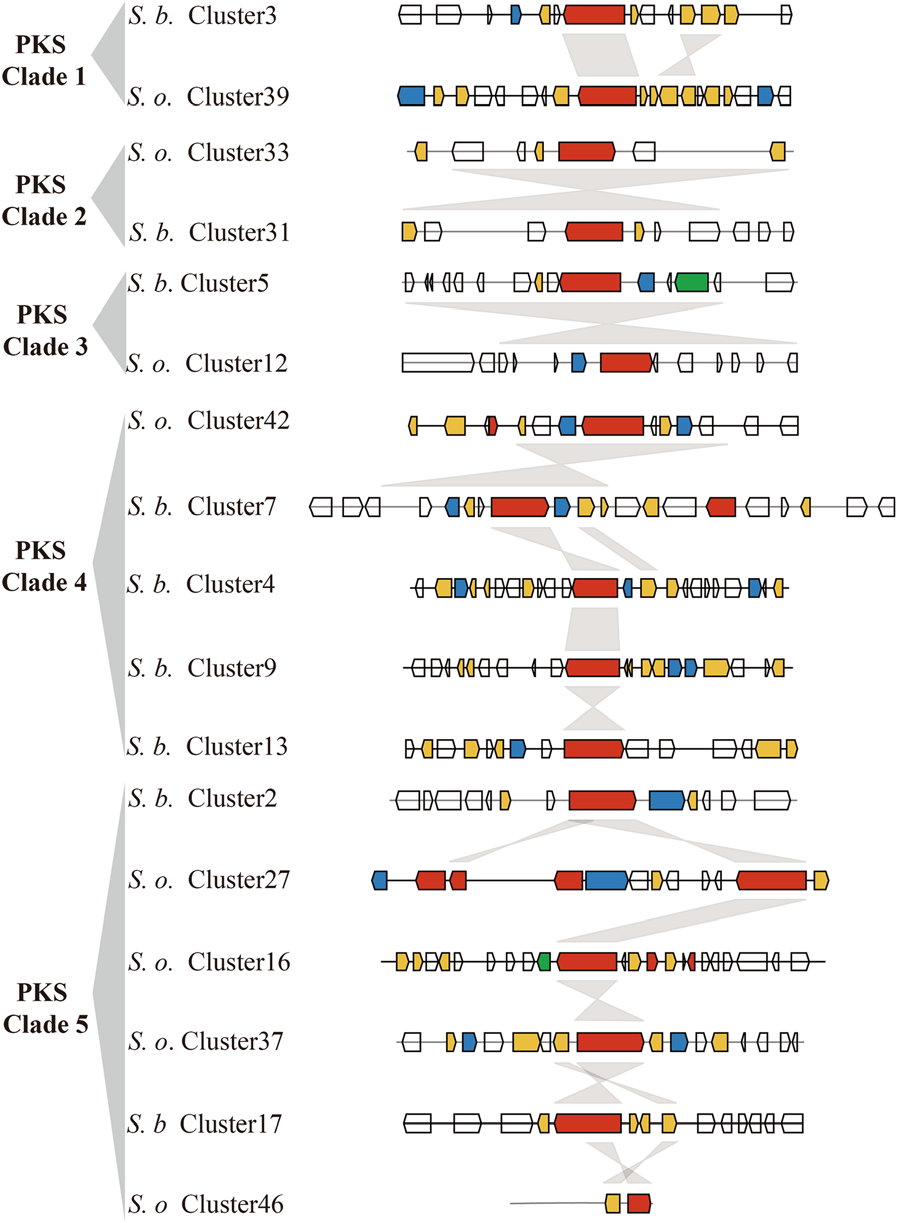
Synteny analysis of PKS gene clusters in *S. brachiariae* and *S. oryzae*. Red arrow: Secondary metabolite synthase gene; yellow arrow: Tailoring enzyme gene; blue arrow: Transport or resistance related gene; green arrow: Regulator gene

**Fig. 10:**
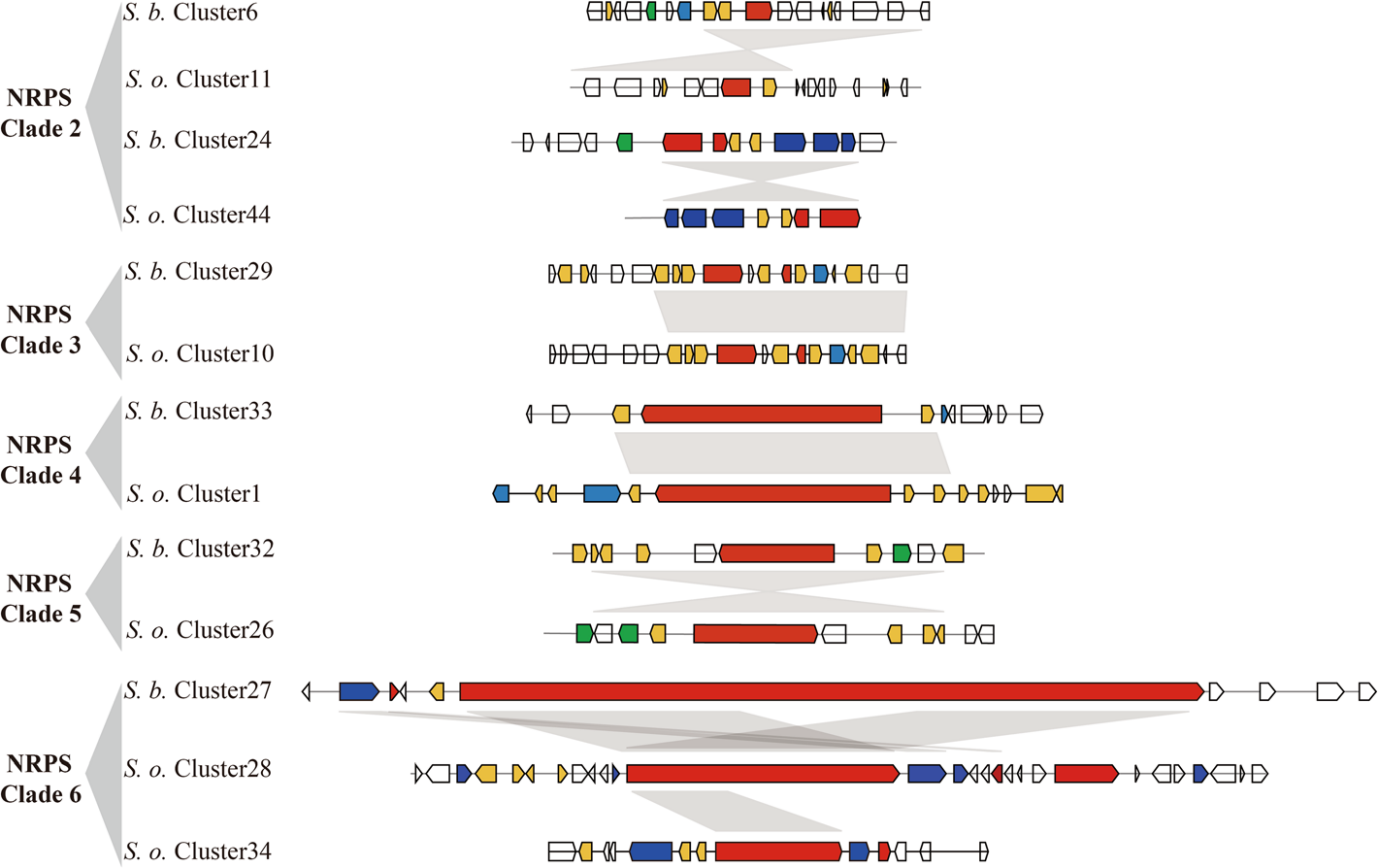
Synteny analysis of NRPS gene clusters in *S. brachiariae* and *S. oryzae*. Red arrow: Secondary metabolite synthase gene; yellow arrow: Tailoring enzyme gene; blue arrow: Transport or resistance related gene; green arrow: Regulator gene

**Fig. 11:**
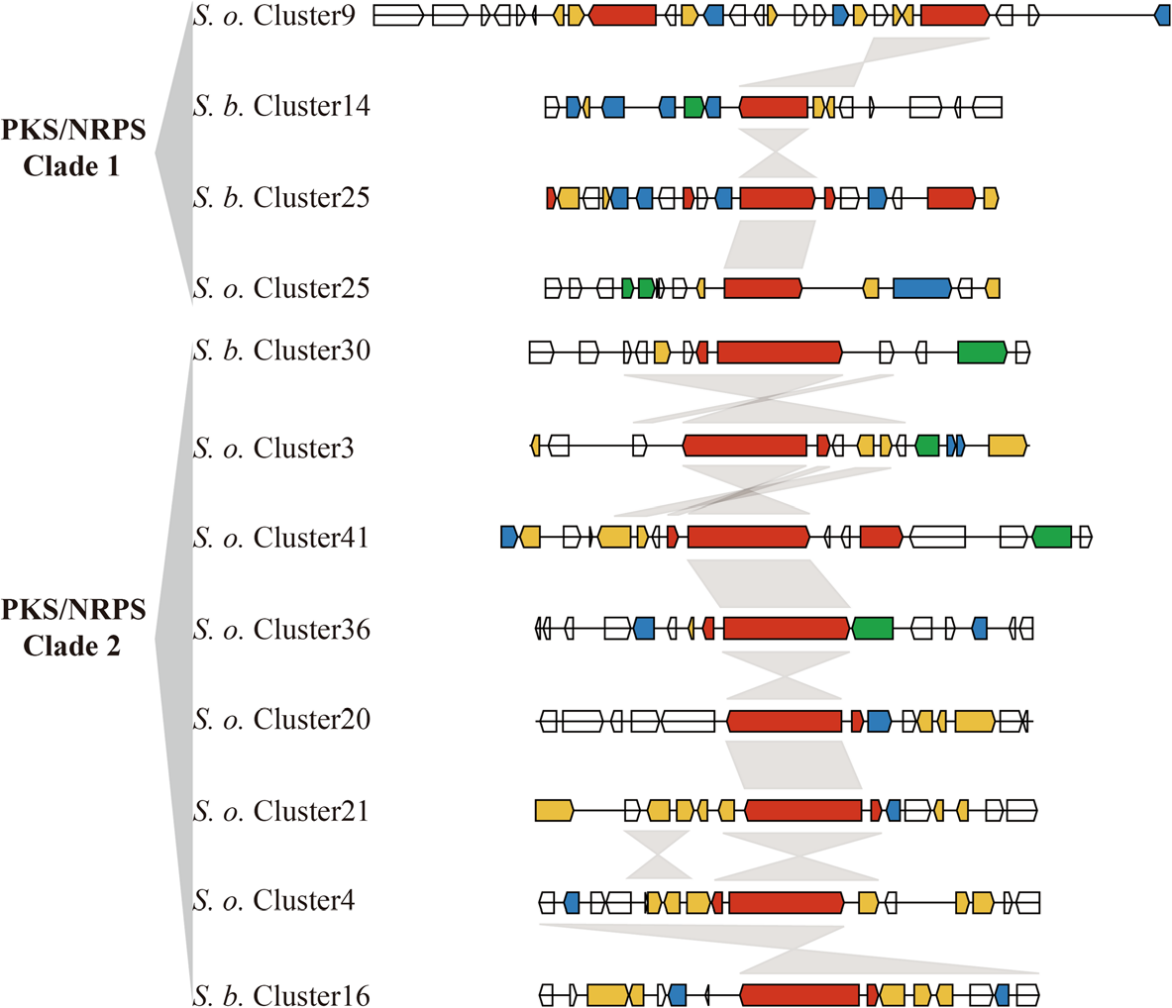
Synteny analysis of PKS/NRPS gene clusters in *S. brachiariae* and *S. oryzae*. Red arrow: Secondary metabolite synthase gene; yellow arrow: Tailoring enzyme gene; blue arrow: Transport or resistance related gene; green arrow: Regulator gene

Except for backbone synthesis genes whose enzymatic products produce a core metabolite, such as NRPSs and PKSs, a contact biosynthetic gene cluster (BGC) also contains genes involved in product modification, transport, and transcription regulation [15]. Closely related species may share some specific BGCs, but the identity and total number of BGCs can vary widely between them [25]. To analyze the differences between BGCs from *S. brachiariae* and *S. oryzae*, synteny of BGCs in which the backbone genes are in the same clade were analyzed. Figures 9, 10, 11 showed that 42 of 48 (87.5%) tested PKSs, NRPSs, and PKS/NRPSs gene clusters had some synteny between *S. brachiariae* and *S. oryzae,* and the remaining six clusters (12.5%) did not have any synteny. Among the gene clusters with synteny, we found six pairs of clusters with the same gene content (*S. brachiariae* Cluster31–*S. oryzae* Cluster 33, *S. brachiariae* Cluster 7–*S. oryzae* Cluster 42, *S. brachiariae* Cluster 24–*S. oryzae* Cluster 44, *S. brachiariae* Cluster 29–*S. oryzae* Cluster 12, *S. brachiariae* Cluster 32–*S. oryzae* Cluster 26, and *S. brachiariae* Cluster 16–*S. oryzae* Cluster 4), just 25% (12 of 48) of all the analyzed clusters. Three pairs of clusters (*S. brachiariae* Cluster 5–*S. oryzae* Cluster 12, *S. brachiariae* Cluster 6–*S. oryzae* Cluster 11,and *S. brachiariae* Cluster 33–*S. oryzae* Cluster 1) had basically the same gene content except for one or two genes, and accounted 12.5% (6 of 48) of all the analyzed clusters. The remaining clusters with synteny had similar core genes but totally different contents of other genes, which accounted for 50% of the tested clusters. Taken together, 37.5% of PKSs, NRPSs, and PKS/NRPSs gene clusters between *S. brachiariae* and *S. oryzae* had good synteny, while most of these clusters, 62.5%, had poor or no synteny. This result suggested a big difference between the secondary metabolisms of *S. brachiariae* and *S. oryzae*. This data also indicated that *S. brachiariae* could potentially synthesize a variety of unknown-function SMs, which may play an important role in adaptation to its endophytic lifestyle and antifungal activity.

## Discussion

The *Sarocladium* genus presently encompasses 17 species, including three phytopathogens, two endophytes, six opportunistic human pathogens, and one mycoparasite [2, 3]. Studies on *Sarocladium* have been focused on plant and human pathogenic species because of their danger to crops and humans. To date, only three species have genome sequence data and all of them cause plant or human diseases. Among them, *S. oryzae* is the phytopathogenic species whose whole genome has been sequenced and published [6]. The other two species are human pathogens; the genome sequence of *S. strictum* has been determined, but no paper has been published (GCA_900290465.1) and the *S. kiliense* data comprises only raw sequence reads [37]. In addition to pathogenic species, endophytic *Sarocladium* species have been found in different kinds of plants, such as *Brachiaria brizantha*, maize, and water mint [4, 38, 39]. Two endophytic species, *S. brachiariae* and *S. zeae*, have demonstrated substantial biocontrol potential on phytopathogens [40]. However, the lack of a determined genome sequence has restrained further research on the life cycle and biocontrol mechanism of endophytic species. In the present study, we focused on deciphering the genomic basis underlying the differences between endophytic and pathogenic *Sarocladium* species. To this end, we generated a reference genome for the endophytic species, *S. brachiariae*, and performed a comparative analysis of genomic data between *S. brachiariae* and the phytopathogen, *S. oryzae*.

In our former result, we established the new *Sarocladium* species, *Sarocladium brachiariae*, based on morphological characteristics together with large subunit (LSU) and ITS rDNA sequences [4]. In the present study, we firstly analyzed the phylogenic relationship of *S. brachiariae* with eight fungal species that have close relationships with *Sarocladium* according to Summerbell et al [3]. The Maximum Likelihood phylogeny tree generated based on single-copy orthologous proteins supports our former result and *S. brachiariae* was classified with *S. oryzae* with 100% bootstrap support. The phylogenomic analysis presented in this study is also largely in agreement with typical phylogenetic studies that sampled species of *Acremonium*, *Sarocladium,* and other genera belonging to the Hypocreales and generated phylogenic relationships from Maximum Likelihood analysis via RAxML VI-HPC of ribosomal RNA gene (LSU + SSU) datasets [2, 3].

Plant cell walls are the first and major barriers to infection by fungal pathogens, and are also the main component of plant biomass. To penetrate into plant cells or use plant cells as a carbon source, plant parasitic fungi secrete a cocktail of hydrolytic enzymes known as carbohydrate-active enzymes (CAZymes) [41]. Zhao et al. identified and compared the full repertoires of CAZymes from symbiotic, biotrophic, hemibiotrophic, necrotrophic, and saprophytic fungi, and performed a comprehensive comparison of the distribution and abundance of CAZyme families. Based on their results, symbiotic and biotrophic fungi contain less CAZymes and less plant cell wall degrading enzymes than necrotrophic and hemibiotrophic fungi, such as GH61, GH78, PL1, and PL3. Most obviously, symbiotic and biotrophic fungi lack GH6 enzymes, which have endoglucanase and cellobiohydrolase activities [10]. In the present study, we identified that the endophytic fungus *S. brachiariae* has more CAZymes and plant cell wall degradation-related CAZymes than the phytopathogen *S. oryzae*.

In addition, four GH6 enzymes were identified in *S. brachiariae*. Recently, four endophytic fungi (*Hypoxylon* sp. CI4A, *Hypoxylon* sp. EC38, *Hypoxylon* sp. CO27 and *Daldinia eschscholzii* EC12) were reported to be rich in CAZymes and could deconstruct plant cell walls to produce mycodiesel. These four strains all harbor two GH6 enzymes [42]. *Pochonia chlamydosporia* is a soil fungus with a multitrophic lifestyle combining endophytic and saprophytic behavior. Aranda-Martinez et al. showed that this strain also contains significant amounts of CAZymes involved in plant cell wall degradation [43]. On the basis of these results, we would like to propose the following two explanations: (1) endophytic fungi need a certain amount of cocktail of CAZymes to destroy plant cell wall before colonizing host plants or using plant cell wall polysaccharides as nutrients; (2) *S. brachiariae* might have a multitrophic lifestyle and turn to necrotrophic or hemibiotrophic under some circumstances [44–47].

Fungal cell walls, mainly composed of chitin and β-(1,3)-glucan, are also an important barrier to protect fungi against invasion by mycoparasitic fungi or bacteria. Lysis of the cell wall is one of the key steps during mycoparasitism, and mycoparasitic fungi usually poss an expanded set of fungal cell wall degradation-related CAZymes, such as GH18 and GH75 [14, 17]. *Trichoderma* species are mycoparasitic fungi and are used as biocontrol agents against plant pathogenetic fungi. *Trichoderma* have an expanded repertoire of chitinases (GH18), chitosanases (GH75), and β-(1,3)-glucanases (GH55). In the present study, we also noted that *S. brachiariae* possess 33.3% more secreted fungal cell wall degradation-related CAZymes than *S. oryzae*, especially chitinases (GH18) and β-(1,3)-glucanases (GH55, GH16) (Table 3). This result could partially explain the antifungal activity of *S. brachiariae*.

Phytotoxins are low-molecular-weight secondary metabolites capable of disturbing the vital activity of plant cells or causing their death at concentrations below 10 mM. Phytopathogenic fungi are best known as phytotoxin producers [48]. Rice pathogenic *S. oryzae* produces the phytotoxins helvolic acid and cerulenin, both of which cause necrosis and mimic the disease symptoms of sheath rot caused by *S. oryzae* [16, 49]. *S. oryzae* uses these two phytotoxins to change cell permeability, leading to leakage of electrolytes before invading the host tissue [18, 34]. Cerulenin was discovered in the 1960s as antibiotic because it could inhibit fatty acid synthesis in fungi; however, its biosynthesis process remains unclear [50]. Helvolic acid is synthesized by different fungi and its biosynthetic gene cluster (BGC) has been determined in *A. fumigatus* and *M. anisophilae*. The BGC responsible for helvolic acid synthesis contains one oxidosqualene cyclase, one 3-ketosteroid-Δ1-dehydrogenase, one short-chain dehydrogenase/reductase, two acyltransferases, and four CYP5081 family cytochrome P450 genes [19, 20]. Based on de novo sequencing data, Hittalmani et al. identified nine genes related to helvolic acid biosynthesis in *S. oryzae* Saro-13 strain using BLASTP searches of the *S. oryzae* proteome against *A. fumigatus* protein sequences [6]. These nine genes were spread across the whole genome and did not form a common BGC; however, this might have been caused by the poor assembly result (5856 contigs). Based on the antiSMASH analysis, we identified the BGC responsible for helvolic acid synthesis in *S. oryzae* on assembly GCA_001972265.1. The analysis identified that eight continuous genes form the BGC for helvolic acid in *S. oryzae*. Compared with the known BGCs in *A. fumigatus* and *M. anisophilae*, the newly found helvolic acid BGC contains one fewer acyltransferase gene. The structural arrangement of the *S. oryzae* BGC is more similar to that of *M. anisophilae* than to that of *A. fumigatus*. Lv et al. determined the function of each gene and the encoded protein reaction sequences by reconstitution of the nine genes from the BGC of *A. fumigatus* one by one in *A. oryzae* NSAR1. In their study, they proposed that the two acyltransferase had different active site, Held1 (the first acyltransferase) accounts for the acetylation of C-6 hydroxyl group, and Held2 (the second acyltransferase) acts on the C-12β hydroxyl group [35]. However, BGC in *S. oryzae* just has one acyltransferase gene that shares 69% homologous with the Held1 gene. Therefore, we propose that the biosynthetic process of helvolic acid in *S. oryzae* is a little different to the known pathway and the newly identified acyltransferase could act on both C-6 and C-12β hydroxyl groups.

Gene loss is a widespread source of genetic variation that can cause adaptive phenotypic diversity [51]. In plant pathogenic and symbiotic fungi, losses of genes encoding CAZymes, secondary metabolite enzymes, and enzymes in the nitrogen and sulfur assimilation pathways enable biotrophs to adapt to plant hosts by avoiding recognition by the plant defense systems [52]. The best-known gene loss in phytopathogens is the absence of avirulence genes. Loss of avirulence genes could disable the resistant genes in the host plants and enable avirulent isolates to become virulent to phytopathogens, such as *AVR-Pita* in *M. oryzae*, *AvrLm1* in *Leptosphaeria maculans* and *NIP1* in *Rhynchosporium secalis* [53–55]. Using antiSMASH, we identified 44 BGCs in *S. oryzae*, including those encoding NRPSs, PKSs, PKS/NRPS hybrid, terpenes, and others. Meanwhile, in *S. brachiariae*, we found only 34 BGCs, 10 fewer than in *S. oryzae*. KOG distribution analysis also gave the same result, *S. brachiariae* had 53 fewer proteins belonging to “Secondary metabolites biosynthesis, transport and catabolism” than *S. oryzae*. These two strains share the same number of NRPSs; however, although numbers of the PKSs and PKS/NRPS hybrid are very different, the total number of PKSs and PKS/NRPS hybrid is similar. The biggest difference between the BGCs of these two *Sarocladium* sp. is the number of BGCs for terpenes. *S. brachiariae* (seven terpene BGCs) has 46% fewer than *S. oryzae* (13 terpene BGCs). Interestingly, the helvolic acid BGC is included in these six lost terpene BGCs. We further performed manual BLASTP searches of the *S. brachiariae* proteome against helvolic acid synthesis related proteins in *S. oryzae*. Based on the BLASTP result, there is one oxidosqualene cyclase and one 3-ketosteroid-Δ1-dehydrogenase encoding gene in the *S. brachiariae* genome; however, these two genes are located far from each other. In addition, we did not find any gene encoding a cytochrome P450 of the CYP5081 family in *S. brachiariae*. Therefore, the endophytic fungus *S. brachiariae* does not possess the genetic resources to synthesize helvolic acid. Helvolic acid is the major phytotoxin synthesized by *S. oryzae*; therefore, we speculated the lack of helvolic acid-related biosynthetic genes are the adaptive for *S. brachiariae* to form a mutualistic relationship with the host plant as an endophyte.

In plant-associated fungi, non-ribosomal peptides and polyketides have different functions, such as phytotoxins, mycotoxins, and antibiotics. These two different kinds of secondary metabolites are separately synthesized by NRPSs and PKSs, which are both large and multimodular enzymes [56]. The occurrence of horizontal gene transfer, gene duplication and loss, domain acquisition, and point mutations, mean that NRPS and PKS genes are generally recognized as rapidly evolving gene classes in fungi. Fast evolution leads to few clearly identifiable orthologs between species and highly discontinuous distributions, even between closely related species [23, 24]. However, within these two fast evolving enzymes, the ketosynthase (KS) domain and adenylation (A) domain have relatively conserved amino acid sequence in PKSs and NRPSs, respectively. Based on sequences of these two domains, Kroken et al. and Bushley et al. analyzed the phylogenomic relationships of known PKSs and NRPSs, separately [23, 36]. A few studies on secondary metabolites of *Sarocladium* sp. have focused on terpene phytotoxins, but none have investigated NRPSs and PKSs in *Sarocladium* sp. [6, 17, 34]. Based on the Maximum Likelihood phylogenetic trees generated from the amino acid sequences of KS or A domains in *S. brachiariae* and *S. oryzae*, we found that PKSs, NRPSs, and PKS/NRPS with similar domain structures could be clustered together, with high bootstrap support (Figs. 7 and 8). This indicated that the two compared species share a close evolutionary relationship. In addition, we found a high level of gene duplication, especially in PKS Clade 4 (one PKS from *S. oryzae*, four PKSs from *S. brachiariae*) and PKS/NRPS Clade 2 (two PKS/NRPSs from *S. brachiariae*, six PKS/NRPSs from *S. oryzae*) (Figs. 7 and 8). Carbone et al. reported secondary metabolite biosynthetic gene duplication in *Aspergillus*, in which many copies of aflatoxin biosynthetic genes were found in the genome. They suggested the gene duplication drives the evolution of gene clusters involved in secondary metabolites synthesis [57]. This may have resulted the differences in BGCs between *S. brachiariae* and *S. oryzae*.

However, core genes are not sufficient to synthesize secondary products, they need the help of other genes around them, which constitute a contact biosynthetic gene cluster (BGC). Based on their phylogenetic relationship, we analyzed the syntenic relationships of BGCs in which core genes were clustered into one clade. The results indicated that just 37.5% of tested clusters had good synteny, 50% only shared similar core genes, and 12.5% had no synteny. The BGC analysis also indicated that gene duplication only happened for core genes. For example, in PKS Clade 4, *S. oryzae* Cluster42 had good synteny with *S. brachiariae* Cluster7, while the other three *S. oryzae* clusters had poor synteny and only had similar PKS genes. This suggested that gene duplication is responsible for the formation of new BGCs in the *Sarocladium* genus. Lind et al. examined BGC variation in 66 strains of *A. fumigatus*. They found that the BGCs were highly distinct even in strains belong to one species and identified five different types of variants [25]. Our results are consistent with those of Lind’s study, and based on their theory, BGCs variants between *S. brachiariae* and *S. oryzae* should mainly belong be single nucleotide polymorphisms, indels, whole gene cluster polymorphisms, and gene content polymorphisms. BGC variants result in changes in the corresponding metabolites; therefore, we speculated that secondary metabolome of *S. brachiariae* differ markedly from that of *S. oryzae*. Thus, the *S. brachiariae* genome might encode protein responsible for the synthesis of a variety of unknown-function secondary metabolites, possibly related to its adaptation to an endophytic lifestyle and antifungal activity.

## Conclusion

The *Sarocladium* genus contains different kinds of fungi, such as phytopathogens, endophytes, opportunistic human pathogens, and mycoparasites However, to date, only the genome of the phytopathogen *S. oryzae* has been sequenced and published [6]. In the present study, we reported the gapless whole genome sequence of *S. brachiariae*, the first genome sequenced from an endophytic fungus of the *Sarocladium* genus. The whole genome sequencing and de novo assembly revealed that the genome of *S. brachiariae* comprises 31.86 Mb and the G + C content is 52.04%. Comparative genomics analysis revealed that *S. brachiaria* had 14.9% more plant cell wall degradation related CAZymes to *S. oryzae*, and 33.3% more fungal cell wall degradation related CAZymes. The expanded fungal cell wall degradation related CAZymes might be the reason why *S. brachiaria* shows antifungal activity. Based on the antiSMASH analysis result, we identified a contact biosynthetic gene cluster for helvolic acid in *S. oryzae* for the first time. However, we found no gene cluster related to helvolic acid biosynthesis or a gene encoding a cytochrome P450 belonging to the CYP5081 family, which are necessary for helvolic acid biosynthesis, in the genome of *S. brachiariae*. This indicated that the endophytic fungus *S. brachiariae* could not synthesize the phytotoxin helvolic acid. Including the missing helvolic acid BGC, *S. brachiaria* had seven fewer terpene gene clusters compared with those in *S. oryzae*, which might be an adaptation method for its endophytic lifestyle. Synteny analysis of PKS, NRPS, and PKS/NRPS gene clusters between *S. brachiariae* and *S. oryzae* revealed that just 37.5% of these clusters have good synteny, while 63.5% have no or poor synteny. This indicated that the *S. brachiariae* could potentially synthesize a variety of unknown-function secondary metabolites, which may play an important role in adaptation to its endophytic lifestyle and antifungal activity.

These findings will form the basis for further experimental studies on the endophytic fungus *S. brachiariae*.

## Methods

### Culture and genomic DNA and RNA extraction

Endophytic fungi *Sarocladium brachiariae* HND5 (China General Microbiological Culture Collection Center, CGMCC 2192) was isolated and maintained in our laboratory. Fungi was grown on potato dextrose agar medium and incubated at 28 °C for 7 days. Mycelia were harvested and DNA was extracted from grounded mycelia using a modified cetyltrimethylammonium bromide (CTAB) method, as described previously [58]. For RNA extraction, mycelia were harvested from 7-day-old PDA plate and potato dextrose broth culture incubated for 7 days. Mycelia was immediately flash frozen in liquid nitrogen and TRNzol Universal RNA extraction kit (Tiangen, Beijing) was used for total RNA extraction. Agarose gel electrophoresis, a NanoDrop 1000 spectrophotometer (Thermo, USA), and a Qubit fluorometer (Thermo, USA) were used to analyze the integrity, quality, and concentration of total DNA and RNA, respectively.

### Genome sequencing and assembly

DNA was randomly sheared into fragments with an average size of 20 kb using a Covaris g-TUBE. DNA damage and the ends of the sheared DNA were then repaired. SMRTbell templates were obtained by ligating the blunt hairpin adapters to the ends of the repaired fragments, followed by the addition of exonuclease to remove failed ligation products. Before annealing the sequencing primer and binding the polymerase to SMRTbell templates, the quality of library was assessed using an Agilent 2100 Bioanalyzer High Sensitivity Kit (Agilent, USA). SMRT cells were sequenced using the PacBio RS II sequencing platform (Pacific Biosciences, Nextomics Biosciences, Co., Ltd., Wuhan). After filtering out the sequencing adapters and low-quality sequences, clean data (filtered reads: 4.92G, sequencing depth: 191×) were obtained and then assembled using CANU (https://canu.readthedocs.io/en/latest/#) with default parameters [59]. The assembly result was adjusted using Arrow [27] and the integrity of assembly was evaluated using BUSCO [60].

### RNA sequencing and data analysis

RNA sample was sequenced with paired-end, 150-bp reads on Illumina novaseq 6000 system (Nextomics Biosciences, Co., Ltd., Wuhan). The sequencing reads were mapped to the *Sarocladium brachiariae* genome using the TopHat 2.1.1 with default parameters [61].

### Genome annotation

Protein coding genes were annotated using a combination of two different methods: (1) Augustus and Genscan were used to de novo predict protein coding genes by constructing models; (2) GeneWise was used to predict protein coding genes by homology analysis with known protein sequences from related species related species *(Magnaporthe oryzae*, *Fusarium oxysporum*, *Pochonia chlamydosporia*, *Claviceps purpurea* and *Verticillium dahlia*) [62, 63]. EVidenceModeler (EVM) was then used to compute the weighted consensus gene structure annotations [64]. After removing genes with transposable elements using TransposonPSI [65], we obtained the final gene sets.

Multiple databases, including Swiss-Prot, NR, KEGG, and COG were used to make functional annotations for the predicted gene models, using BlastP with E-values ≤1^*e*-5^ [66–68]. BLAST searching against the Rfam database was used to predict non-coding RNAs, such as rRNAs, snRNAs, and miRNAs [69]. RNAmmer and tRNAscan-SE were also used to predict rRNAs and tRNAs, respectively [70, 71].

### Repetitive sequences analysis

Repetitive sequences were identified and analyzed using different methods. Four computer applications were used to identify transposable elements, including the database-based software RepeatProteinMasker and RepeatMasker, and two de novo pieces of software, RepeatModeler (http://repeatmasker.org/RepeatModeler/) and LTRfinder [72–74]. Tandem Repeats Finder was used to analyze tandem repeat sequences and MIcroSAtellite was used to detect the microsatellite DNA (1–6 bp) [75, 76].

### Analysis of orthologous gene families in Sordariomycetes fungi

Gene families were analyzed using orthoMCL [77] (E-values ≤1^*e*-5^) by comparing proteins from *S. brachiariae* with those from other Sordariomycetes fungi: *S. oryzae* (GenBank: GCA_001972265.1), *A. chrysogenum* (GenBank: GCA_000769265.1), *F. graminearum* (GenBank: GCA_000240135.3), *F. oxysporum* (GenBank: GCA_000149955.2), and *M. oryzae* (GenBank: GCA_000002495.2).

### Phylogenetic analysis and synteny analysis

Based on the orthologous gene families analysis, single copy orthologous gene groups were chosen for further phylogenetic analysis. Gblocks [78] (with default parameters) was used to remove divergence and ambiguously aligned blocks from the alignment to obtain a better CDS file. The maximum-likelihood tree was constructed using RaxML with the GTRGAMMA model and 100 bootstrap replicates to infer the phylogenetic relationship of *S. brachiariae* to other Sordariomycetes fungi (*S. oryzae*, *A. chrysogenum*, *F. graminearum*, *F. oxysporum*, *Coniochaeta ligniaria* (GenBank: GCA_001879275.1), *Verticillium dahlia* (GenBank: GCA_000952015.1), and *Pochonia chlamydosporia* (GenBank: GCA_001653235.2) [30]. MUMmer software was used to perform the genome-wide synteny analysis, and synteny dot-plot were generated using mummerplot [32].

### Pathogenicity genes analysis

The *S. brachiariae* proteome was used to make a BLASTP search (E-values ≤1^*e*-5^) against the Pathogen-Host Interaction (PHI) (http://www.phi-base.org) database to identify fungal pathogenicity genes [79].

### Carbohydrate-active (CAZy) enzymes analysis

The *S. brachiariae* proteins were used to make a BLASTP search(E-values ≤1e-5) against the Carbohydrate Active Enzymes database (http://www.cazy.org/) [7]. The results were catalyzed like Glycoside Hydrolases (GHs), Glycosyl Transferases (GTs), Polysaccharide Lyases (PLs), Carbohydrate Esterases (CEs), Carbohydrate-Binding Modules (CBMs), and Auxillary Activities (AAs) as described in CAZy database.

### Cytochrome P450 analysis

The cytochrome P450 gene family classification (E-values ≤1^*e*-5^, identity > 40%) in *S. brachiariae* was performed using the CYtochrome P450 Engineering Database (version6.0)(https://cyped.biocatnet.de/).

### Secretome analysis

The prediction of the refined *S. brachiariae* and *S. oryzae* secretome was based on the procedure described by Brown and colleagues for *Fusarium graminearum* [80]. SignalP (http://www.cbs.dtu.dk/services/SignalP/) was used to predict signal peptides and cleavage sites of *S. brachiariae* and *S. oryzae* proteins [81]. Proteins with a Singal P D-score = Y were analyzed for subcellular location with Target P v1.1 (http://www.cbs.dtu.dk/services/TargetP/) [82]. And proteins with a Target P Loc = S were scanned for transmembrane spanning regions using TMHMM (TMHMM v2.0; http://www.cbs.dtu.dk/services/TMHMM/) and all proteins with 0 TMs or 1 TM, if located in the predicted N-terminal signal peptide, were kept. Proteins with glycosylphosphatidylinositol (GPI) anchor were predicted by big-PI [83] (http://mendel.imp.ac.at/gpi/gpi_server.html). Localization of the remaining proteins without GPI-anchor were predicted with ProtComp using the LocDB and PotLocDB databases (ProtComp v9.0; http://www.softberry.com/berry.phtml?topic=protcompan&group=programs&subgrous=proloc) and proteins predicted as extracellular or unknown were kept for next analysis. Proteins with no methionine at start or mature peptide less than 20 amino acids were discarded. WolfPSort (https://wolfpsort.hgc.jp/) [84] were used to analyze the remaining proteins and proteins with extracellular score > 17 were kept in the final secretome databases.

### Secondary metabolite gene cluster analysis

Putative polyketide synthases (PKS), non-ribosomal peptide synthases (NRPS) genes, PKS-NRPS hybrids, and their modules of different domains were identified via searching in the antiSMASH database (fungal version) (https://fungismash.secondarymetabolites.org/) with default settings [85].

A Maximum Likelihood tree was generated with amino acid sequences of A or KS domains using MEGA version 6.0 with the Wheland and Goldman (WAG) mode [86]. A domains and KS domains sequences used for Maximum Likelihood tree construction were listed in Additional file 6 and Additional file 7. GATA was used to perform synteny analysis of gene clusters, with default settings [87]. Genes found in PKS, NRPS and PKS/NRPS gene clusters were annotated based on BLASTN search results and results were listed in Additional file 4 (*S. brachiariae*) and Additional file 5 (*S. oryzae*).

### PCR validation of in silico gene prediction result of *S. brachiariae*

22 genes were picked from 22 secondary gene clusters of *S. brachiariae* for PCR validation of its in silico gene prediction result. These genes and their primers were listed in Additional file 2: Table S9. TaKaRa Taq DNA polymerase (Takara Biomedical Technology, Beijing) was used for PCR amplification according to product protocol. A touchdown PCR program was used for PCR amplification: 95 °C 2 min; 95 °C 20 s; 65 °C 20 s (− 1 °C per cycle, ramp 2 °C); 70 °C, 40 s; go to step 2 for 20 cycles; 70 °C, 10 min. 1% agarose gel electrophoresis was used for PCR products analysis and target DNA bands were cut and purified for Sanger sequencing (Huada Gene Technology, Shenzhen).

## Supplementary information

**Additional file 1: Figure S1.** Agarose gel electrophoretogram of PCR amplification products of 22 genes chosen for validation of in silico gene prediction result of *S. brachiariae*. Line 1–22: WHWLZ0913, WHWLZ1690, WHWLZ1900, WHWLZ1931, WHWLZ2227, WHWLZ2744, WHWLZ2917, WHWLZ4323, WHWLZ5125, WHWLZ5511, WHWLZ5935, WHWLZ6101, WHWLZ6305, WHWLZ7443, WHWLZ7561, WHWLZ7986, WHWLZ8202, WHWLZ8477, WHWLZ9395, WHWLZ9449, WHWLZ9463 and WHWLZ9474.

**Additional file 2: Table S1.1.** BUSCO analysis of *S. brachiariae* scaffolds assembly; **Table S1.2.** Statistics of RNAseq data; **Table S1.3.** Mapping rate of NGS data; Table 2. RNA statistics of *S. brachiariae*; **Table S3.** Repeative sequence statistic of *S. brachiariae*; **Table S4.** Gene annotation statistic of *S. brachiariae*; **Table S5.1.** KOG distribution of predicted proteins from *S. brachiariae*; **Table S5.2.** KOG distribution of predicted proteins from *S. oryzae*; **Table S6.1.** Secreated proteins of *S. brachiariae* and *S. oryzae*; **Table S6.2.** CAZymes distribution of predicted proteins from *S. brachiariae*; **Table S6.3.** CAZymes distribution of predicted proteins from *S. oryzae*; **Table S7.1.** SM Clusters coordinates of *S. brachiariae* scaffolds assembly; **Table S7.2.** SM Clusters coordinates of *S. oryzae* scaffolds assembly; **Table S8.** Cytochrome P450 distribution of predicted proteins from *S. brachiariae*; **Table S9.** Genes chosen for PCR validation of in silico gene prediction result of *S. brachiariae* and primers design.

**Additional file 3: Table S1.** Helvolic acid biosynthetic gene cluster found in *S. oryzae*; **Table S2.** Possible proteins involved in helvolic acid biosynthesis found in *S. brachiariae*.

**Additional file 4.** Annotation (BLASTp) of genes in PKS, NRPS and PKS/NRPS gene clusters of *S. brachiariae*.

**Additional file 5.** Annotation (BLASTp) of genes in PKS, NRPS and PKS/NRPS gene clusters of *S. oryzae*.

**Additional file 6.** A domain sequences used for phylogenetic analysis.

**Additional file 7.** KS domain sequences used for phylogenetic analysis.

**Additional file 8. **Sanger sequencing results of the PCR products of the 22 genes chosen for PCR validation of in silico gene prediction result of *S. brachiariae*. (TXT 20 kb)

## Data Availability

The genome assembly of *S. brachiariae* generated and analyzed during this studyhas been deposited at DDBJ/ENA/GenBank under the accession RQPE00000000. The version described in this paper is version RQPE01000000. The raw sequence reads are deposited in NCBI SRA database under accession number SRR8202370. The raw sequence reads of RNAseq are deposited in NCBI SRA database under accession number SRR9289321.

## Abbreviations

A: Adenylation
ACP: Acyl Carrier Protein
antiSMASH: Antibiotics & Secondary Metabolite Analysis Shell
BGC: Biosynthetic Gene Cluster
C: Condensation
CAZyme: Carbohydrate-active Enzyme
CE: Carbohydrate Esterase
CWEDs: Cell Wall-degrading Enzymes
CYP: Cytochrome P450
DH: Dehydratase
GH: Glycoside Hydrolase
KR: Ketoreductase
KS: Ketosynthase
KSTD: 3-ketosteroid-Δ1-dehydrogenase
MAT: Malonyl-CoA:acyl Carrier Protein Transacylase
NRPS: Non-ribosomal Peptide Synthetases
OSC: Oxidosqualene Cyclase
PKS: Polyketide Synthases
PL: Polysaccharide Lyases
SDR: Short-chain dehydrogenase/reductase
SM: Secondary Metabolite
T: Thiolation
TC: Terpene Cyclases
