# Supplementary figures and images for: Genomic characteristics and comparative genomics analysis of the endophytic fungus Sarocladium brachiariae
Source: BMC Genomics. 2019 Oct 28;20:782. doi: 10.1186/s12864-019-6095-1 (PMC6819638; doi:10.1186/s12864-019-6095-1)

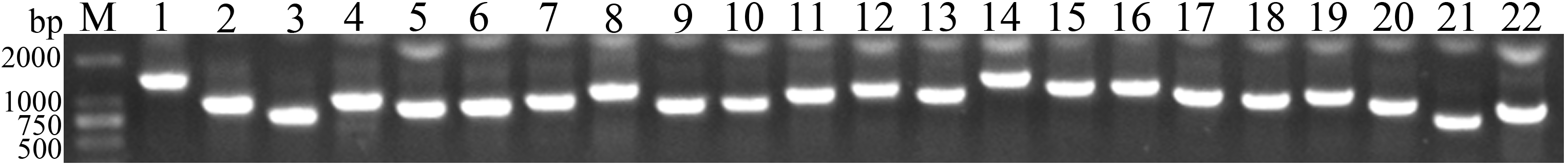

Supplement: Supplementary file 1 — Additional file 1: Figure S1. Agarose gel electrophoretogram of PCR amplification products of 22 genes chosen for validation of in silico gene prediction result of S. brachiariae. Line 1–22: WHWLZ0913, WHWLZ1690, WHWLZ1900, WHWLZ1931, WHWLZ2227, WHWLZ2744, WHWLZ2917, WHWLZ4323, WHWLZ5125, WHWLZ5511, WHWLZ5935, WHWLZ6101, WHWLZ6305, WHWLZ7443, WHWLZ7561, WHWLZ7986, WHWLZ8202, WHWLZ8477, WHWLZ9395, WHWLZ9449, WHWLZ9463 and WHWLZ9474. [file 12864_2019_6095_MOESM1_ESM.tif]
